# In-Line Detection of Clinical Mastitis by Identifying Clots in Milk Using Images and a Neural Network Approach

**DOI:** 10.3390/ani13243783

**Published:** 2023-12-08

**Authors:** Glenn Van Steenkiste, Igor Van Den Brulle, Sofie Piepers, Sarne De Vliegher

**Affiliations:** Department of Internal Medicine, Reproduction and Population Medicine, Faculty of Veterinary Medicine, Ghent University, 9820 Merelbeke, Belgiumsofie.piepers@ugent.be (S.P.); sarne.devliegher@ugent.be (S.D.V.)

**Keywords:** deep learning, clinical mastitis, automated milking systems, image recognition, clots in milk

## Abstract

**Simple Summary:**

The study focused on improving the detection of clinical bovine mastitis, the inflammation of the udder in cows as a response to intramammary infection, which can be identified by the presence of clots in the milk. Currently, automated milking systems do not detect this important disease very accurately. To address this, we developed a clots detection program using a neural network. This neural network was trained to recognize clots in milk samples from dairy cows by using a large number of pictures of milk filter socks, some with and some without clots. These pictures were divided into different sets for training, validating, and testing the program, respectively. The settings of the neural network were optimized using a genetic algorithm. The program’s interpretations were explained using a method called integrated gradients. The program was found to be 100% accurate in identifying clots in the test pictures. This suggests that the method could be very useful for automatically checking for clinical mastitis on dairy farms, although further field validation through integration into the existing systems is needed.

**Abstract:**

Automated milking systems (AMSs) already incorporate a variety of milk monitoring and sensing equipment, but the sensitivity, specificity, and positive predictive value of clinical mastitis (CM) detection remain low. A typical symptom of CM is the presence of clots in the milk during fore-stripping. The objective of this study was the development and evaluation of a deep learning model with image recognition capabilities, specifically a convolutional neural network (NN), capable of detecting such clots on pictures of the milk filter socks of the milking system, after the phase in which the first streams of milk have been discarded. In total, 696 pictures were taken with clots and 586 pictures without. These were randomly divided into 60/20/20 training, validation, and testing datasets, respectively, for the training and validation of the NN. A convolutional NN with residual connections was trained, and the hyperparameters were optimized based on the validation dataset using a genetic algorithm. The integrated gradients were calculated to explain the interpretation of the NN. The accuracy of the NN on the testing dataset was 100%. The integrated gradients showed that the NN identified the clots. Further field validation through integration into AMS is necessary, but the proposed deep learning method is very promising for the inline detection of CM on AMS farms.

## 1. Introduction

From an economic viewpoint, mastitis is one of the most important diseases in dairy cows [1,2,3,4,5], due to its effects on animal health and the subsequent losses in milk production, as well as the need to discard abnormal milk or milk from diseased cows (European Union Directive EC/853/2004 and US Food and Drug Administration Grade A pasteurized milk ordinance). Depending on the study, the cost of each clinical mastitis (CM) varies between USD65 and 930 [2,3,4]. The early detection of CM can reduce both the economic impact and the long-term impact on cow health and welfare [6,7].

Many dairy farms are transitioning to automated milking systems (AMSs), with around 38,000 units installed worldwide in 2017 [8]. When using AMSs, there are fewer opportunities for the farmer to detect CM in individual animals. The current AMS incorporates a variety of milk monitoring and sensing equipment, but the sensitivity and specificity of its CM detection capabilities remain relatively low, with most systems having a sensitivity between 47 and 90% and a specificity between 56 and 99% [9,10]. For reference, the International Standards Organization (ISO) describes a standard target of 90% sensitivity and 99% specificity for the detection of abnormal milk (ISO/FDIS (Final Draft International Standard) 20966 [11]), Annex C (Automatic Milking Installations—Requirements and Testing). Most of these sensor systems try to detect mastitis by measuring and analyzing indirect parameters, such as (but not limited to) electrical conductivity, somatic cell count, milk flow rate, changes in milk color, milk yield per hour or quarter, and cow activity [7,10,12,13,14,15].

A typical symptom of CM is the presence of clots in the milk during pre-milking, which has been proposed as the gold standard for the detection of CM [16,17]. Therefore, we propose to use an in-line camera to detect such clots in the filter after the pre-milking phase. A similar sensor has been proposed in the past but was limited in its capabilities of detecting the clots on the filter, as it was developed to score the quality of the milk and needed to be adapted for instances of different detriments occurring in the milk [18]. Another study proposed to measure clot density in quarter milk samples, which could be useful in monitoring milk quality and clinical mastitis [19]. The researchers used in-line filters to collect quarter milk samples and visually scored the clot density, based on the coverage of the filter area. They showed that high scores clustered within certain cows and periods, suggesting a potential threshold for detecting abnormal milk. The objective of the present study was the development and evaluation of a neural network (NN) capable of detecting such clots on pictures of the filters of the milking system after the pre- and/or milking phase at the cow level.

## 2. Materials and Methods

### 2.1. Experimental Data

The data for this study were generated by adding debris (including straw, hay, manure, bedding material, mud, teat sealer, calcium, and/or flies) and/or clots from used milk filters of AMSs to milk, before passing this milk through a circular milk filter (Universal Hygia Favorit filters, Universal dairy equipment) mounted in a PVC tube. Debris and clots were collected from 40 filters, half of which had clots and half of which did not. These samples were gathered from multiple AMSs and various cows. A vacuum pump provided suction for pulling the milk through the filter. The filters were painted blue for better visualization of the clots. An iPhone 6s was mounted in the PVC pipe to take a photo with the flashlight after each pass of milk. In total, 696 pictures were taken from filters with clots, and 586 pictures from filters without clots.

### 2.2. Image Analysis

For the training dataset, the images without clots were randomly resampled using the built-in python random.uniform function to obtain an even number of images with and without clots for balancing the NN weights. In total, 1676 images were used for training, validating, and testing. During the training of the NN, the images were augmented by random rotations, flipping, rescaling, zooming, and shearing (Table 1), using the Keras ImageDataGenerator function.

**Figure 1:**
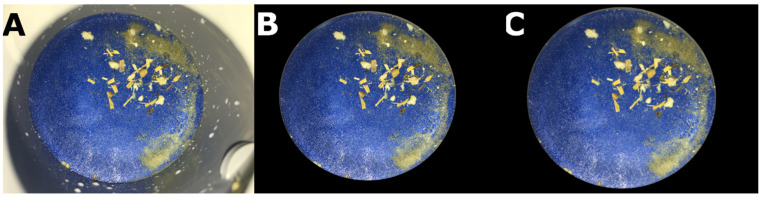
Image pre-processing steps on filter image after the passage of milk with clots. Panel (**A**): original image taken with a resolution of 4032 × 3034 pixels. Panel (**B**): image after applying a black mask on the region of the PVC pipe. Panel (**C**): resulting image used for the neural network after rescaling to 500 × 500 pixels.

To avoid the NN learning features from outside of the filter, e.g., milk spatters on the PVC pipe, the PVC pipe was removed from the picture using OpenCV v4.1. The image was then rescaled to 500 × 500 pixels as the NN input (Figure 1).

**Figure 2:**
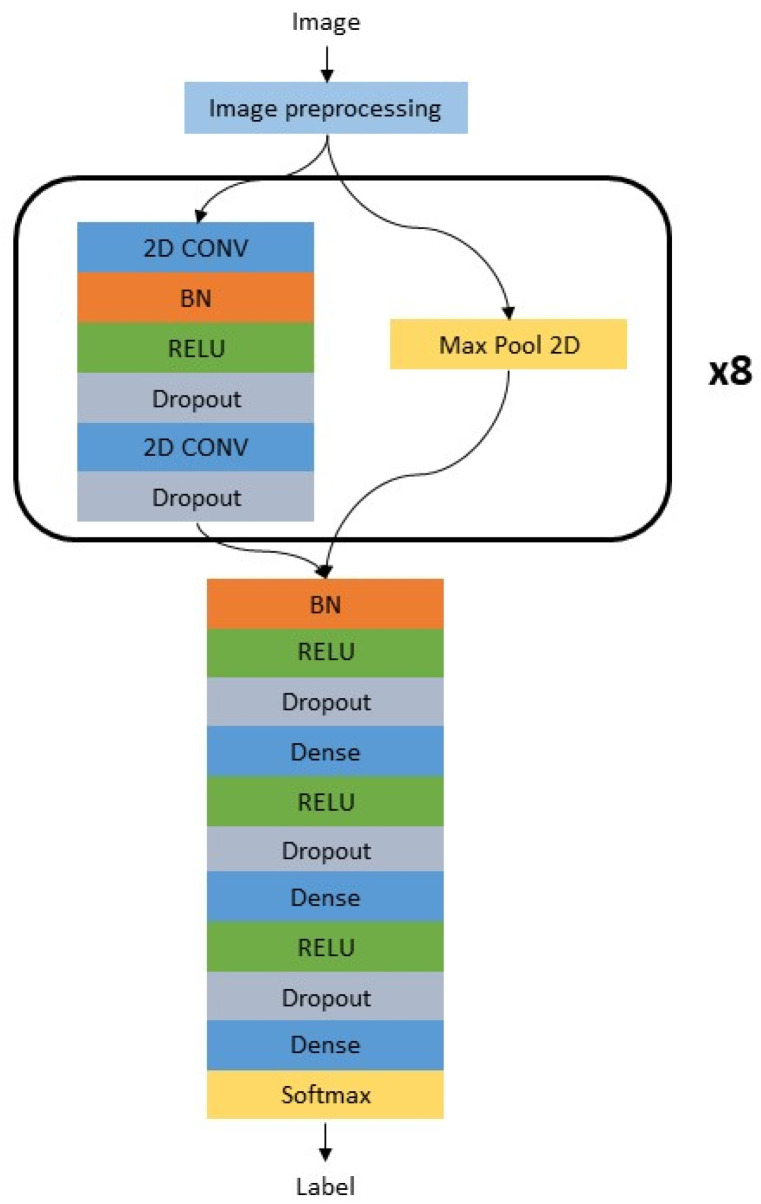
High-level architecture of the neural network. CONV: convolutional block; BN: batch normalization; RELU: rectified linear activation unit; Max Pool 2D: Max pooling operation; Dense: fully connected layer; Softmax: Softmax activation block with the two different output classes.

A genetic algorithm was used to optimize the hyperparameters of the NN (Figure 2) based on the validation dataset. A non-dominated sorting genetic algorithm II (NSGA-II) was used, with a population size of 20 and 10 optimization generations, using the accuracy as the fitness value [20]. The training of each child NN of the algorithm was ended after 50 epochs or when the training stopped improving for three epochs. Optimization was used for the following hyperparameters: the number of filters, the width of convolution and subsampling for each convolutional layer, the number of neurons for each fully connected layer, L2 regularization, and the dropout used for training (Table 2).

**Figure 3:**
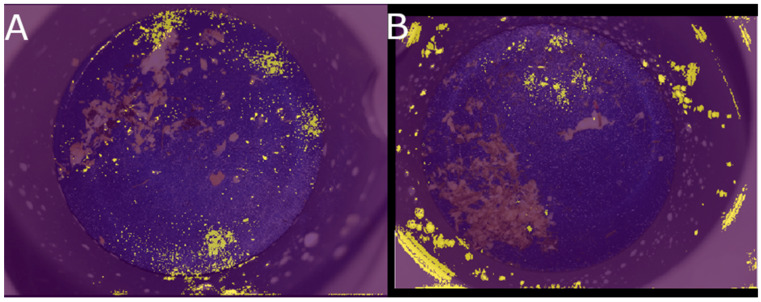
Visualization of the integrated gradients by an attribution mask over the original input image. Yellow indicates a high attribution of the indicated pixels to the output label of the neural network (NN). Panel (**A**) shows the integrated gradients of the currently used NN. Here the pixels (around) the clots attribute the most to the output label. Panel (**B**) shows the integrated gradients of a NN which was trained on images where the PVC pipe was not removed from the image. This ‘cheating’ NN used the milk spats on the PVC pipe to identify if this was an image with clots.

For training, the SoftMax cross entropy was used to calculate the loss with the Adam optimizer and with the default parameters at a learning rate of 0.0001 for updating the weights of the network [21]. The network was trained using parameters optimized by the genetic algorithm, with each epoch evaluated using a validation dataset. This process was repeated for 100 epochs, selecting the best network weights based on validation results, to avoid overfitting the training dataset. The testing dataset was employed for statistical analysis. The network was built in Keras with a TensorFlow v2.0.1 backend [22]. The batch size was set to 16. Afterwards, the integrated gradients of the NN were calculated and compared to a completely black baseline image to obtain an insight into the input–output behavior of the neural network. The attribution of the input pixels to the output labels was projected as a mask over the input image using the OpenCV toolbox (Figure 3).

### 2.3. Statistical Analysis

The dataset was randomly divided into a training subset containing 60% of the data, a validation dataset containing 20%, and a holdout subset containing 20%, which was not used for tuning the model; this resulted in 1006 training, 335 validation, and 335 holdout images. The following metrics were calculated on the holdout dataset: accuracy, positive and negative predictive values, specificity, and sensitivity.

## 3. Results

The accuracy, specificity, positive and negative predictive values, and sensitivity results of the NN on the testing dataset were 100% (Table 3). The integrated gradients showed that the NN identified the clots and accurately distinguished the clots from other materials including straw, hay, manure, bedding material, mud, teat sealer, calcium, and flies.

## 4. Discussion

The accuracy, positive- and negative predictive value, specificity, and sensitivity of the NN on the holdout dataset are a clear improvement, in comparison with other milk-based CM detection methods. Since the proposed method can reliably detect clots in foremilk, this method could be a feasible approach to detect CM in AMSs. Since clots during foremilking are considered the gold standard for detecting CM, the current approach has the potential to be a more reliable CM detection implementation for AMSs, in comparison with current CM detection sensors, which try to detect CM with other parameters, including electrical conductivity, L-lactate dehydrogenase, milk color, and somatic cell counting [9,16]. Many different sensors and algorithms have been proposed for the detection of CM, but, thus far, none of the published CM detection methods achieved the ISO target of at least 80% sensitivity and 99% specificity [23].

One of the main benefits of the current approach is the extremely high accuracy. The main frustration of dairy farmers is the current high number of false alarms made by the available CM detection sensors in AMSs [24]. Due to the low prevalence of (severe) CM, the majority of alerts will indeed be false positives, leading to a potential underreporting of CM cases, as farmers may stop investigating all alerts [23,24,25]. With the proposed sensor, the detection and management of severe mastitis on AMS farms could be significantly improved, reducing the number of false-positives and ensuring that all cases of severe CM are accurately identified and treated and that milk is separated. Even if the practical implementation of the current sensor would not have an accuracy/precision of 100%, a NN, as we have proposed, can be adapted in order to maximize the accuracy, by penalizing false positive results during the training process or by calculating the receiver operating characteristic curve and setting a manual threshold for the minimal required specificity [26]. An additional benefit of using a NN is their robustness for the presence of a variety of detriments (such as straw, manure, udder or tail hair, sawdust, sand, and the remainders of internal teat sealants) on the image, without the need to retrain the algorithm for every possible detriment. This is in contrast with the previous proposed work, in which the fuzzy logic algorithm had to be adapted to recognize the different detriments [18]. If environmental changes (e.g., change in filter type) would overwhelm the robustness of the NN, the weights of the NN could be updated on-site using transfer learning to adapt to the new environment [27]. If the farmer receives multiple false positive results from the sensor, he could initiate an update of the algorithm remotely based on the incorrectly classified images without the need to re-engineer the algorithm. In addition, the proposed NN approach could also use an optional reference image to differentiate and track animals with varying mastitis cases. The algorithm identifies changes in clots by comparing new images with the reference image, which would not be possible with the fuzzy logic algorithm.

If a 3D representation of the filter could be created, e.g., by adding a second camera for 3D stereovision, the NN could also be adapted to calculate the volume of the clots. Calculation of the animal’s clot volume allows us to monitor diseased animals with CM over time and to estimate the severity of the disease and clinical recovery, and, hypothetically, even the likelihood of a bacteriological cure. For example, if the clot volume of a diseased animal is decreasing between consecutive milkings, the animal is likely to be recovering. If few clots are present, increasing milking frequency may suffice as treatment, reducing antibiotic use. On the other hand, treatment can be necessary when many clots in milk are detected. Monitoring the dynamics, as well as the gradual increase in the densities, of the clots on the filter over a period of time could also be a valuable tool to identify cows with chronic mastitis [19].

The integrated gradients showed that the NN identified the region of the clots as an input feature (Figure 3). Neural networks are notorious for having a “black box” approach. It is difficult to attribute the prediction of an NN to its input features and, thus, to know why an NN tells us which pixels of an image are responsible for picking a certain label [28]. The aim of explainable artificial intelligence (AI) is to understand the input–output behavior of NNs. One such explainable AI method is the integrated gradients approach, in which the attribution of each pixel is calculated by summing the gradients (a partial derivative of each variable, while all others are held constant) of the network on different points at the path between a baseline image (e.g., a black image) to the actual input image (e.g., the image of the filter with clots). If the PVC pipe had not been removed from the input images, the integrated gradients would have clearly shown that the NN had learned to recognize the different milk spatter patterns instead of recognizing the clots.

The current study encountered limitations due to the relatively small dataset, which lacked diversity. Although image augmentation techniques were applied to introduce variability, such approaches do not compare to the larger, more complex datasets typically utilized in deep learning studies [29]. Furthermore, the consistency of the recording setup throughout the study, i.e., exclusively using an iPhone’s flash for illumination, has left the model’s robustness to alternate lighting conditions untested; this is a notable concern since, in field conditions, lighting can be inconsistent and obstructed. Consequently, the findings presented here should be interpreted as preliminary, serving as an exploratory investigation into the application of deep learning for mastitis detection. It is recommended that future research be conducted with more extensive datasets gathered from field conditions in AMSs, to thoroughly evaluate the model’s performance and practicality.

While our proposed milk sensor can greatly enhance the detection and management of severe mastitis on AMS farms, it is important to remember that, due to the sudden onset of severe CM, sensor information based solely on changes in milk and measurements collected during milking may not be sufficient for all cases [23]. Cows with severe CM may not visit the AMS. Therefore, a combination of several sensor-based (including activity sensors) and AMS-based indicators may have to be incorporated to meet the necessary demands. It is worth noting that other proposed methods already work at the quarter level [10], and by incorporating additional filters and cameras into the AMS at the quarter, we could potentially enhance the performance of our proposed detection system to also achieve this level of monitoring. The performance of a sensor-based detection system may also be enhanced by the combination of sensor-based or automatic milking-based monitoring systems with additional monitoring strategies, such as visual observations. Therefore, while our sensor offers significant advancements, it should be used in conjunction with other tools and strategies for optimal results.

## 5. Conclusions

The current paper proposed an inline CM detection sensor for AMSs. Further validation and integration on farms with AMSs are necessary, but the proposed method appears to be very promising for the accurate inline detection of CM on AMS farms.

## Figures and Tables

**Table 1:** Image augmentation parameters used by the Keras ImageDataGenerator function during training of the neural network.

Augmentation Type	Value
Random rotation	60°
Random shift in width	10%
Random shift in height	10%
Random zoom	100–130%
Horizontal flip probability	50%
Vertical flip probability	50%
Shear range	20°
Fill mode	nearest

**Table 2:** Hyperparameters selected by the genetic algorithm after 10 optimization generations. For the convolutional layers, the first number indicates the residual block and the second number indicates the convolutional layer within the block. Abbreviations: Conv: convolutional layer.

Layer	Hyper Parameter	Value
All	Dropout	0.2
Conv 1.1	Filters	8
	Kernel size	4
	Subsampling	2
Conv 1.2	Filters	8
	Kernel size	1
	Subsampling	1
Conv 2.1	Filters	16
	Kernel size	4
	Subsampling	1
Conv 2.2	Filters	16
	Kernel size	1
	Subsampling	1
Conv 3.1	Filters	24
	Kernel size	4
	Subsampling	1
Conv 3.2	Filters	24
	Kernel size	1
	Subsampling	1
Conv 4.1	Filters	32
	Kernel size	4
	Subsampling	1
Conv 4.2	Filters	32
	Kernel size	1
	Subsampling	1
Conv 5.1	Filters	40
	Kernel size	4
	Subsampling	1
Conv 5.2	Filters	40
	Kernel size	1
	Subsampling	1
Conv 6.1	Filters	48
	Kernel size	4
	Subsampling	1
Conv 6.2	Filters	48
	Kernel size	1
	Subsampling	1
Conv 7.1	Filters	48
	Kernel size	4
	Subsampling	2
Conv 7.2	Filters	48
	Kernel size	1
	Subsampling	1
Conv 8.1	Filters	48
	Kernel size	4
	Subsampling	1
Conv 8.2	Filters	48
	Kernel size	1
	Subsampling	1
Dense 1	Number of neurons	64
	L2 regularization	0.1
Dense 2	Number of neurons	32
	L2 regularization	0.01

**Table 3:** Coherence matrix of results.

	Predicted Negative	Predicted Positive
True negative	182	0
True positive	0	153

## Data Availability

Data are available upon reasonable request to the corresponding author.
